# Pairwise Measures of Causal Direction in the Epidemiology of Sleep Problems and Depression

**DOI:** 10.1371/journal.pone.0050841

**Published:** 2012-11-30

**Authors:** Tom Rosenström, Markus Jokela, Sampsa Puttonen, Mirka Hintsanen, Laura Pulkki-Råback, Jorma S. Viikari, Olli T. Raitakari, Liisa Keltikangas-Järvinen

**Affiliations:** 1 IBS, Unit of Personality, Work and Health Psychology, University of Helsinki, Helsinki, Finland; 2 Centre of Expertise for Human Factors at Work, Finnish Institute of Occupational Health, Helsinki, Finland; 3 Helsinki Collegium for Advanced Studies, University of Helsinki, Helsinki, Finland; 4 Department of Medicine, Turku University Hospital and University of Turku, Turku, Finland; 5 Department of Clinical Physiology, Turku University Hospital and Research Centre of Applied and Preventive Cardiovascular Medicine, University of Turku, Turku, Finland; Imperial College London, United Kingdom

## Abstract

Depressive mood is often preceded by sleep problems, suggesting that they increase the risk of depression. Sleep problems can also reflect prodromal symptom of depression, thus temporal precedence alone is insufficient to confirm causality. The authors applied recently introduced statistical causal-discovery algorithms that can estimate causality from cross-sectional samples in order to infer the direction of causality between the two sets of symptoms from a novel perspective. Two common-population samples were used; one from the Young Finns study (690 men and 997 women, average age 37.7 years, range 30–45), and another from the Wisconsin Longitudinal study (3101 men and 3539 women, average age 53.1 years, range 52–55). These included three depression questionnaires (two in Young Finns data) and two sleep problem questionnaires. Three different causality estimates were constructed for each data set, tested in a benchmark data with a (practically) known causality, and tested for assumption violations using simulated data. Causality algorithms performed well in the benchmark data and simulations, and a prediction was drawn for future empirical studies to confirm: for minor depression/dysphoria, sleep problems cause significantly more dysphoria than dysphoria causes sleep problems. The situation may change as depression becomes more severe, or more severe levels of symptoms are evaluated; also, artefacts due to severe depression being less well presented in the population data than minor depression may intervene the estimation for depression scales that emphasize severe symptoms. The findings are consistent with other emerging epidemiological and biological evidence.

## Introduction

Statistical measures of causality have been introduced for cross-sectional data [1]–[4]. Despite their obvious usefulness for the study of epidemiology, serious attempts to apply these methods have been rare or negligible; this is perhaps in part due to over-generalizations made from the well-known fact that a cross-sectional correlation does not imply causality. What is true for correlation, however, does not generalize to all aspects of distributions; it can be shown that information in higher moments of distribution does sometimes allow causal inferences [1]–[4]. This study applies pairwise causality measures to an acute problem in epidemiology: estimation of the direction of causality between depression and sleep problems. Acknowledging the small amount of real-data testing, we first estimate causality in a case that should be logically evident: parents’ socioeconomic status should cause that of the offspring’s rather than the other way around. Then real data on depression and sleep problems is investigated. Finally, a simulation study is conducted in order to further support the findings. We next explicate why the issue of causality between depression and sleep problems is a difficult and acute research problem in epidemiology.

Sleep problems have rapidly climbed among the leading health problems in western societies. Point prevalence estimates of insomnia vary between 6% and 48%, depending on the definition and sample/country [5], [6]. Sleep problems are a great burden to the individual and costly for the society, because poor sleep can decrease work performance [7] and increase the risk of non-fatal and fatal accidents [8], [9]. Sleep problems predict cause-specific work disability, and are associated with subsequent disabling mental and physical illnesses [10]. Over three-fold risk of disability retirement due to all causes have been attributed to frequent sleep problems [11], and recent studies show that insomnia with objectively measured short sleep duration is associated with poor cognitive performance and increased mortality [12], [13]. Depression is another major public health problem. It is projected to be the second largest cause of the global burden of disease by the year 2030 [14]. Life-time prevalence of depression has been estimated to be approximately 16% [15], also with high variability between countries [16]. Depression leads to various social role impairments [15], and it has a recurrence rate of up to 85% [17].

Complaints of poor sleep quality are estimated to occur in 50% to 90% of diagnosed cases of depression [16], [18], and there is higher prevalence of depression in patients with obstructive sleep apnea [19]. Although sleep disorders are traditionally included as one of the symptoms of depression, this view has been challenged. Different aspects of sleep display various links with depression that are clearly physiological [18]–[22]. The issue of causality between depression and sleep problems, however, has remained obscure [18]–[20].

Sleep problems often precede the onset of melancholic/depressed mood [23]–[26]. Temporal order of appearance is a classical sign of causality, and it has been suggested that sleep problems may actually cause depression [24], [27], [28]. However, sleep problems may also reflect a prodromal symptom of depression [25], which is why temporal precedence alone is insufficient to confirm causality. Depression, as currently measured, is a heterogeneous set of affect-related and somatic symptoms [29]–[31]. Thus, it would be unfeasible to assume that all of the symptoms would emerge at the onset of depression [30]. Rather, various symptoms may emerge one at time, until a significant amount of depression can finally be diagnosed. The order of appearance may reflect the sensitivity of the underlying homeostatic process for disturbances as well as causality; hence, longitudinal sampling may be insufficient to prove causality. Yet, knowledge of the correct causality is vital for efficient development of theories and interventions.

These considerations make evident that an efficient cross-sectional measure of causality would be useful in determining whether sleep problems cause depression or depression causes sleep problems. Recent work in computational statistics has shown that the use of information in the higher-order (non-Gaussian [32]) moments of population distribution does allow the determination of causality in certain situations: the causal relation between variables is assumed to be linear, the error terms need to be *non-*Gaussian (i.e., distributed according to some other than the Normal/Gaussian distribution) and the causal connection must conform to an *acyclic* graph [1]–[3], [33]. In principle, the acyclicity requirement, implying that reciprocal effects are not allowed, can be relaxed [4]. A further assumption is that an unobserved confounder does not cause both the variables [1]–[3]; the extent to which this needs to hold can be evaluated via computer simulation. Jointly these assumptions are known as the Linear, Non-Gaussian Acyclic Model (LiNGAM) [1].

The important assumption of non-Gaussian distribution should logically hold for population distributions of a depression and sleep problems scores, as both the variables should be skewed towards the majority of people having little issues and only a minor part at the severe end of the continuums. Furthermore, recent studies suggest that depressive symptoms form a causal network of symptoms that directly influence each other, instead of reflecting a single latent causal antecedent [30]. This suggests that the association between sleep problem symptoms and other depressive symptoms is not fully confounded by a latent third factor, but a detectable dominant causal direction may exist.

We apply several pairwise measures of causality in order to gain information regarding causality between depressed moods and sleep problems in the community-based samples of Young Finns and Wisconsin Longitudinal studies. Such measures have provided reasonable information about causality in content domains relating to physical system and sociological data; for example, within a system of variables including father’s education and occupation, number of siblings, son’s education and occupation, and son’s income, only one out of the five causal connections simultaneously estimated by the algorithm was illogical [2]. This study also estimates the pairwise causal direction for parents’ and their offspring’s socioeconomic status (SES), in a hope that recovering the logically self-evident outcome builds further trust for the statistical methodology (empirical benchmark). Provided that sensible estimates of causal direction can be drawn from the mentioned cross-sectional questionnaire data, the same methods may also help in elucidating the causal relationship between sleep problems and depressed mood.

**Figure 1:**
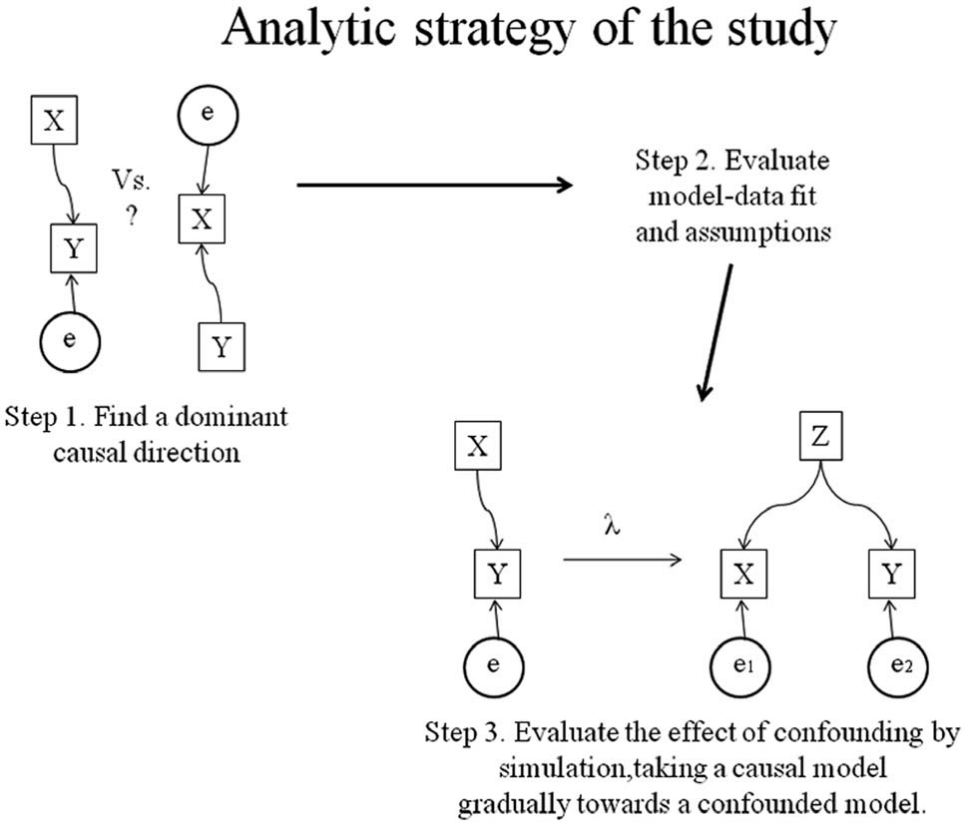
Analytic strategy of the study. First, a causality algorithm is applied to infer whether the variable Y is a weighted sum of the variable X and a residual term e (X causes Y), or vice versa. Second, assumptions of the applied causal model are evaluated. Third, a simulation study probes the model’s sensitivity for assumption violations that are difficult to evaluate directly; most importantly, the impact of the partial confounding on the algorithms ability to recognize causal association is evaluated.

Most testing for causality algorithms has been performed using simulated data where the ‘ground truths’ are known for certain and diverse conditions can be tested. The estimation efficiency depends on the specific algorithm and on several data parameters [1]–[3]; therefore we also perform some simulations in a situation similar to our data at hand (simulation-based benchmark). The methods and results sections are organized 1) by estimation of pairwise causality [1]–[3]; 2) by evaluation of LiNGAM assumptions and fit in the data sets; and 3) by further exploration of the models validity via simulation (Figure 1 sketches the data-analytic flow of the study). The discussion section summarizes our logical conclusions from these steps. Despite the subtleties involved, the causal modeling appeared to provide useful information from a novel analytic angle.

## Materials and Methods

### Participants

Data from two separate population studies were used. First, the Cardiovascular Risk in Young Finns study is an on-going population-based cohort study [34]; its participants have provided a written informed consent, and it has been approved by the ethical committee of the Varsinais-Suomi’s hospital district’s federation of municipalities. Second, data from Wisconsin Longitudinal Study was used; data was initially collected via a telephone interview, after which a questionnaire was mailed to the participants [35]. Informed consent was obtained at the beginning of the telephone interview. All instruments and operations were approved by the Institutional Review Board of the University of Wisconsin-Madison.

The original Young Finns sample consists of 3596 healthy Finnish children and adolescents derived from six birth cohorts, aged 3, 6, 9, 12, 15, and 18 years at baseline in 1980. In order to select a broadly sociodemographically representative sample, Finland was divided into five areas according to locations of university cities with a medical school (Helsinki, Kuopio, Oulu, Tampere and Turku). In each area, urban and rural boys and girls were randomly selected on the basis of their unique personal social security number. The sample has been followed subsequently in 7 data collection waves in 1983, 1986, 1989, 1992, 1997, 2001, and 2007. A detailed description of cohort can be found in an earlier publication [34]. The most recent follow-up in 2007 included questions on sleep as well as questionnaires on depression. Participants who provided full data for both measures were included in this strictly cross-sectional study. Table 1 illustrates the basic characteristics of the sample. In addition, a benchmark analysis of 1348 participants with the required data confirmed that their parents’ socioeconomic status in year 1983 could be estimated as being a causal antecedent to their own socioeconomic status in the year 2007, rather than a causal descendent (see Text S1 for more information about the benchmark SES variables).

**Table 1:** Sample Characteristics and Attrition in the Young Finns Study.

Data for comparisons between modified BDI (1. depression scale) and Sleep problems					
Measure (unit/range)	Study sample		Attrition sample		p-value
Number of participants	1699		1897		
Percentage of males	41.1 %		56.2 %		< .001
	**mean**	**range**	**mean**	**range**	
Age of participants (years)	37.71	30–45	37.20 (n = 1897)	30–45	.002
	**mean**	**s.d.**	**mean**	**s.d.**	
Sleep problems score (1–6)	2.28	1.05	2.31 (n = 463)	1.06	.543
Depression score (1–5)	2.00	0.66	2.14 (n = 333)	0.65	< .001
**Data for comparisons between BDI-II (2. depression scale) and Sleep problems**					
**Measure (unit/range)**	**Study sample**		**Attrition sample**		**p-value**
Number of participants	1687		1909		
Percentage of males	40.9%		56.2%		< .001
	**mean**	**range**	**mean**	**range**	
Age of participants (years)	37.67	30–45	37.24 (n = 1909)	30–45	.011
	**mean**	**s.d.**	**mean**	**s.d.**	
Sleep problems score (1–6)	2.27	1.04	2.34 (n = 475)	1.07	.186
Depression score (0–3)	0.23	0.30	0.54 (n = 328)	0.64	< .001

Note: p-value is from t- or chi-squared test for the difference between the study and attrition samples, and s.d. denotes standard deviation. Attrition sample consists of participants who lacked information either regarding depression or regarding sleep. Some had one but not other, allowing comparison against those with both. For such cases, n denotes sample size for this sub-sample.

The Wisconsin Longitudinal Study [35] is a prospective cohort study of a random sample of 10 317 participants (5326 women, 4991 men) born between 1937 and 1940, and followed since they graduated from Wisconsin high schools in 1957. After baseline data collection in 1957, survey responses were collected in 1964, 1975, 1992, and 2004. The sample is broadly representative of white, non-Hispanic US men and women who completed at least high school education. It is estimated that about 75% of Wisconsin youth graduated from high school in the late 1950s – everyone in the primary sample graduated from high school. A mail questionnaire collected in 1992–93 contained a depression inventory and 3 sleep items [35]. Participants who provided full data in both measures were included in this study. Table 2 illustrates the basic characteristics of this sample.

**Table 2:** Sample Characteristics and Attrition in the Wisconsin Longitudinal Study.

Data for comparisons between modified CES-D (3. depression scale) and Sleep problems					
Measure (unit/range)	Study sample		Attrition sample		p-value
Number of participants	6640		3677		
Percentage of males	46.7 %		51.4 %		< .001
	**mean**	**range**	**mean**	**range**	
Age of participants (years)	53.14	52–55	53.19 (n = 3084)	52–55	< .001
	**mean**	**s.d.**	**mean**	**s.d.**	
Sleep problems score (1–6)	1.24	1.75	0.63 (n = 90)	1.48	.001
Depression score (0–140)	16.40	15.44	23.31 (n = 167)	19.81	< .001

Note: p-value is from t- or chi-squared test for the difference between the study and attrition samples, and s.d. denotes standard deviation. Attrition sample consists of participants who lacked information either regarding depression or regarding sleep. Some had one but not other, allowing comparison against those with both. For such cases, n denotes sample size for this sub-sample.

### Measures

#### Young finns study

For the empirical benchmark test, the causality between parents’ socioeconomic status and that of their offspring was estimated using pairwise measures. The SES variance was a z-score standardized sum of the z-score transformed variables measuring years of education, level of education, and gross income. More details about these variables are provided in the supplementary material (Text S1).

Depression was assessed with two different versions of the Beck’s Depression Inventory (BDI). The first was a modified version in the Young Finns study, representing the second mildest symptom statement of each item of the original BDI [36] as a five-point scale ranging from ‘not at all’ to ‘very much’ [37], [38]. The average of such items provides a measure that samples a larger range of variation for more similar and milder depressive tendencies than the original BDI. We refer to this average as the modified BDI (mBDI). Potentially sleep-related items ‘I get tired faster than before’ and ‘Waking up in morning, I am much more tired than before’ were excluded.

The second version was a slightly modified version of Beck’s Depression Inventory II (BDI-II) [39], [40]. First, because we did comparisons with sleep problems, items that reflected sleep problems were removed (item 16 about increased/decreased amount of sleep and item 20 about subjective feeling of tiredness). Second, because direct comparability with the original sum score is already lost by removal of these items, we used an average of the remaining items (having values 0, 1, 2 or 3) as the total score rather than sum of all items. Despite these small changes, we refer to this latter score as BDI-II. Both measures had 21 items, 19 of which were used here. mBDI (average of 19 items) and BDI-II correlated with coefficient .77 and had respective Cronbach’s alpha reliabilities of .92 and .91. A sensitivity analysis indicated that exclusion of sleep related items did not perceivably affect the results for either scale (Table S1).

Sleep problems were assessed with Jenkin’s scale consisting of four items that assess: difficulties falling sleep, frequent awakenings, troubles staying asleep (including too early waking), and feelings of tiredness and exhaustion after a regular night of sleep [41]. These items were answered with the following six-point precision: 1 = ‘not at all’; 2 = ‘1–3 nights in month’; 3 = ‘1 night in week’; 4 = ‘2–4 nights in week’; 5 = ‘5–6 nights in week’; and 6 = ‘every night’; the average of the four items formed the final measure of Sleep problems. Cronbach’s reliability coefficient alpha for Sleep problems was .77.

#### Wisconsin longitudinal study

Depression was measured with the Center for Epidemiologic Studies Depression Scale [42], a modified version (mCES-D) fully described in the study’s web page [35]. The scale consists of 20 items and describes a level of psychological distress ranging from 0 (the lowest possible) to 140 (the highest possible). The individual items assesses for how many days of the past week the participant felt a given depressive symptom or distress.

Sleep problems were coded with zero if the participant had answered that he or she did not have trouble sleeping in the past six months. Otherwise, it was coded as a sum of two items with a following content: ‘How often have you had trouble sleeping?’ (1 = ‘monthly or less often’; 2 = ‘about once a week’; 3 = ‘daily or more often’) and ‘How much discomfort has trouble sleeping caused you in the past six months?’ (0 = ‘none’; 1 = ‘a little’; 2 = ‘some’; 3 = ‘a lot’).

### Statistical Analyses

#### Pairwise causality estimation

The pairwise causality estimation, as applied here, starts from the assumptions that 1) either sleep problems *x_s_* cause depression or depression *x_d_* causes sleep problems, 2) the causal association is linear, 3) independent residual terms are non-Gaussian (distributed according to some other than the Normal distribution), and 4) there are no confounding variables. This is the Linear, Non-Gaussian, Acyclic Model (LiNGAM). Mathematically it means that for centered/zero-mean variables either.
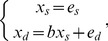
(1)or
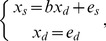
(2)where es and/or ed is a non-Gaussian variable, and b is a constant regression coefficient. The aim of the algorithm is to estimate which one holds, system of equations 1 or system of equations 2, and to estimate the quantitative value of the coefficient b. In these two alternative systems of equations, either depression or sleep problems is an exogenous variable: an exogenous variable is not predicted by other variables in the system, and can be considered as an input to a system of variables. The estimated exogenous variable is causal because the other variable is its function, and it is not a function of the other variable. In other words, manipulations of an exogenous variable lead to changes in the other (endogenous) variable, but manipulations of an endogenous variable do not affect the exogenous variable.

With non-Gaussian variables and the LiNGAM model, all we need to do in order to determine the causality is to estimate which one is the exogenous variable, *x_d_* or *x_s_*, by estimating which one is less dependent on its residuals. In the DirectLiNGAM-algorithm [2], dependency is evaluated using a nonparametric kernel-based estimator [43] of the mutual information [32]. After these differences, LiNGAM is just a linear regression model with a non-Gaussian error term. In the two-variable case, DirectLiNGAM-algorithm is similar to (causal) non-linear correlation [3]; the connection between the methods derives from the pairwise measure of dependency. Causality can also be inferred from more restricted/approximate non-Gaussianity properties of the distributions of *x_d_* and *x_s_* [3]. Text S2 discusses the mathematics of three causality statistics, or three causal non-linear correlations, that were applied here: DirectLiNGAM-based, Skew-based, and Tanh-based (based on hyperbolic-tangent approximation to Likelihood Ratio for distributions with non-Gaussian kurtosis). For each statistic, a positive value signifies causal antecedence of the first argument/variable and a negative value indicates the opposite condition. The DirectLiNGAM-based measure applies the default options of the DirectLiNGAM-algorithm version 1.0; that is, the pairwise causality statistic used by the more general DirectLiNGAM-algorithm [2] (freely provided by authors [44]). The algorithm is implemented for the Matlab^®^ software (Natick, Massachusetts, USA), and was applied in the version 7.10.0. (R2012a). The same software was used for computation of other causality statistics according to equations outlined in the Text S2.

Population sampling, estimation procedures, and partial incorrectness of assumptions can introduce variability to statistical estimates. Totality of variability can be assessed by bootstrapping [45]. One randomly draws *with replacement* several (2000 here) bootstrap re-samples from the original data, all equal to the original in number of observations. Each re-sample is thus drawn from the same underlying distribution, but is not quite the same as the original sample. Relevant estimates are then calculated for each sample and their variabilities over the bootstrap re-samples are assessed. When an estimated solution is unstable, bootstrap standard errors are large or the estimated direction of causality varies for different bootstrap re-samples. Regarding causality results, we provide a percentage for how often in all bootstrap re-samples the causal antecedence is estimated for a given variable by a given causality statistic (Tables 3 and 4). In addition, the median causality statistic over the bootstrap re-samples is reported, together with 95% Bias-corrected and Accelerated bootstrap confidence interval for the statistic [45].

#### Evaluation of LiNGAM assumptions and model-data fit

After estimating the direction of causality using the three pairwise measures, we evaluated the required LiNGAM assumptions (model fit) by assessing 1) the linearity hypothesis for the estimated causal direction, 2) whether the residual distribution was non-Gaussian, 3) and whether it was independent of the exogenous variable. The data was considered to exhibit a linear relationship if the quadratic term in Ordinary Least Squares regression was non-significant, and a scatter plot visually supported the linearity-interpretation. The error-term distribution was considered non-Gaussian if a hypothesis of Gaussian/Normal distribution was rejected by the Lilliefor’s test [46]; visual evaluation was also performed using kernel density estimates (ksdensity-function from Matlab's Statistics-toolbox with default options) and histograms. The independence between Ordinary Least Squares regression residuals and ‘independent’/exogenous variable was visually inspected, statistically tested using a distribution-independent L^1^ test with the suggested four equiprobable partitions [47], and tested with the non-parametric Hoeffding’s test [48].

In principle, the applied L^1^ test of independence assumes that there are no atoms (discreteness) in the data [47], but adding small amount of jitter (low-variance uniform random variable) to observations prior to regression model estimation, thereby removing atoms, did not alter the test result (not shown). P-value was estimated by matching the test-threshold with observed test-statistic using a standard function-minimizer (Matlab’s *fminsearch*-function, i.e., the Nelder-Mead simplex method) with a quadratic loss-function. For the Hoeffding’s test [48], Frank E Harrell’s implementation was applied from version 3.9–3 of Hmisc-package under R-software version 2.13.0 [49]. The test is consistent in the class of distribution functions with continuous joint and marginal probability densities. Our empirical distributions were sums of ordinal items; therefore the robustness of result was verified by adding a low variance (0.1) normal random variable to each observed sum score, and by observing that similar results ensue (not shown).

#### A simulation study

Although pair-wise measures of causality (or nonlinear correlations) are quite robust against measurement error [3], tolerance for all assumption violations has not been directly tested. Furthermore, testing such violations in a simulated situation that approximates the data at hand provides additional confidence to the results at hand. Therefore, we performed a short simulation study as outlined below.

As a preliminary, a continuous probability model was estimated that closely approximated the observed data-distributions by fitting a mixture distribution of four Gaussians [50] to the linear model residual and a (location-shifted) exponential distribution to the independent variable (standard functions from Matlab Statistics-toolbox were used). Fully controlled artificial observations can be drawn/simulated from these probability distributions. A thousand data sets were simulated in each test condition, always with number of observations equal to that in the observed real data (n = 1699, as for the mBDI in Young Finns data). For each test condition, ‘estimation success’ was calculated as a proportion of pairwise causality estimates with ‘correct’ causality; correct is defined below case-wise. The sensitivity for assumption violations was evaluated by plotting the estimation success for each test condition as a function of the degree of assumption violation.

First, the effects of discretization (analogous to ordinal variables) were evaluated by simulating independent-variable values, *x*, and residual values, *e*, from the above-defined distributions, by computing dependent values, *y = βx + e*, from the linear model, and by then imposing a discrete lattice that spans the interval from *x* and *y* variables’ minimum to its maximum. For example, the interval [min(*y*), max(*y*)] was divided to *k* intervals, and all values falling to given interval were set to equal the lower limit of that interval. We tested values of *k* ranging from 2 to 15, setting *β* to equal the Ordinary Least Squares estimate for the real observations. In these test conditions, a correct causality estimate was the one that gave the causal direction corresponding to the underlying simulated continuous model (i.e., *x* → *y*).

Second, the effect of confounding was assessed, where confounding was either *linear* or *proportional*. *Linear* confounding meant that the observations were simulated by giving the underlying model a weight (1–*λ*), and by adding a proportion *λ* from a (simulated) confounded model. Therefore, the dependent variable was of the form *y*_confounded_ *= *(1–*λ*)*βx + λβz + e*, and the independent variable of the form *x*_confounded_ = (1–*λ*)*x + λ*(*βz + e_z_*), where *z* was another variable with the same distribution as for *x*, and *e_z_* was another residual variable similar to *e*. Fifteen test conditions were evaluated, where the degree of confounding (values of *λ*) ranged from 0 to 1: value zero means no confounding in the original model, value one means full confounding by an ‘unobserved’ third variable, and intermediate values of *λ* represent an intermediate degree of confounding (co-existence of discernible causal direction and latent confounding). In *proportional* confounding, the simulated values were not a weighted sum of unconfounded and confounded values, but a proportion *λ* of observations was drawn from the fully confounded model and proportion (1–*λ*) from the fully unconfounded model. In both cases, linear and proportional, four distribution settings were tested: 1) *x* and *z* were exponentially distributed, *e* and *e_x_* from a Gaussian Mixture distribution (“GM residual” in figure legends); 2) the roles were reversed (Exp residual); 3) *x*, *z*, and *e_z_* were exponentially distributed, *e* from a Gaussian Mixture distribution (Different residuals); 4) all variables, *x*, *z*, *e* and *e_z_*, were from a Gaussian Mixture distribution (All GM). In all cases, the residual distributions were translated to have a zero mean. Here, the ‘correct’ estimate is the one that yielded the simulated causal direction (*x → y*) despite partial masking due to confounding; for the fully confounded model, one (the same) direction is arbitrarily chosen, and should yield approximately the proportion ½ for the estimation success.

Third, we demonstrated robustness against Gaussian measurement error in observations by adding a Gaussian random variable to *x* between the computation of *y = βx + e* and the application of causality statistic (correct direction: *x → y*). Fifteen different measurement-error standard deviations were examined. Although, previous simulations have been performed [3], this confirms the error-tolerance in a situation that closely correspond to our data. All the steps of the simulation study, discussed above, were separately performed for all the three causality statistics; that is, for the DirectLiNGAM-based, Skew-based, and Tanh-based statistic.

#### Graded response modeling for depression scale differences

Finally, we wanted to obtain a crude picture regarding the relative depression severity encoded by different scales, and this was possible for mBDI and BDI-II because altogether 1993 Young-Finns-Study participants had answered to both of the scales. A Graded Response Model with the ‘logit’-response function [51]–[53] was fitted simultaneously to the items of the both scales, and sums of the item-informations of the respective scales indicated the relative information per scale that can be plotted as a function of depression severity. It is generally expected that the local independence assumption [51]–[53] does not hold for depression-questionnaire items [30], and therefore the absolute fit of a unidimensional Graded Response Model is bound to be more or less bad. Only the relative information about the differences between mBDI and BDI-II was of interest here, and was judged to warrant reporting as supporting information for the other analyses (the exact code for the procedure is provided as Demos S1); because the result may be of general interest and no direct comparison with sleep problems is involved in the Graded Response Model, all the 21 items of each scale were used.

## Results

### Pairwise Causality Estimates

Table 1 displays the basic characteristics of the Young Finns sample, and the sample that was excluded due to missing data. Table 2 shows the same for the Wisconsin Longitudinal study. Sample correlation between depression and sleep problems was clear in all three data sets (mBDI: r = 0.41 with a 95% confidence interval of (0.37, 0.45); BDI-II: r = 0.39 (0.34, 0.43); mCES-D: r = 0.37 (0.35, 0.39)); the same held for the correlation between parents’ and offspring’s socioeconomic status (SES) in benchmark data (r = 0.41 (0.37, 0.46)). Table 3 summarizes the results from causal analyses between depression (BDI-II or mBDI) and sleep problems in the Young Finns data; also the results for benchmark SES data are reported therein. Table 4 shows the results from causal analysis between depression (mCES-D) and sleep problems in the data from Wisconsin Longitudinal Study.

**Table 3:** Pairwise Causality Comparisons for 2000 Bootstrap Re-samples in Young Finns Data.

	Chosen as cause %		Summary of values	
Method/Statistic	Parents' SES	Offspring's SES	Statistic	95% confidence int.
DirectLiNGAM^b^	100.00	0.00	0.1062	(0.0627, 0.1485)
Skew-based	100.00	0.00	0.0721	(0.0454, 0.1019)
Tanh-based	99.90	0.05	0.0077	(0.0033, 0.0124)
	**mBDI**	**Sleep problems**		
DirectLiNGAM^a^	00.40	99.60	−0.0433	(−0.0747, −0.0090)
DirectLiNGAM^b^	01.40	98.60	−0.0354	(−0.0677, 0.0001)
Skew-based	2.80	97.20	−0.0276	(−0.0565, 0.0009)
Tanh-based	28.50	71.50	−0.0013	(−0.0054, 0.0027)
	**BDI-II**	**Sleep problems**		
DirectLiNGAM^a^	77.65	22.35	0.0213	(−0.0332, 0.0781)
DirectLiNGAM^b^	100.00	0.00	0.1633	(0.0927, 0.2572)
Skew-based	100.00	0.00	0.0913	(0.0457, 0.1507)
Tanh-based	65.95	34.05	0.0011	(−0.0038, 0.0058)

a Non-standardized original variables (not available for SES).

b Standardized variables; Skew- and Tanh-based statistic always require standardization. Second and third column report the percentages of ‘wins’ in the indicated pairwise comparison, whereas the two last columns summarize the statistic implying the result over the 2000 re-samples. SES = socioeconomic status, mBDI = modified Beck’s Depression Inventory; BDI-II = Beck’s Depression Inventory II.

All three pairwise causality statistics easily recognized parents’ SES as a causal antecedent for their offspring’s SES; among the bootstrap re-samples, each statistic is almost always positive (Table 3), signifying that its first argument (parents’ SES) is the causal antecedent of the second (offspring’s SES). Each method therefore recovers the desired for logical result in the empirical benchmark data. Few failures that occurred in the Tanh-/kurtosis-based estimates may be due to fact that skewness rather than kurtosis is the dominant departure from Gaussian distribution for the SES variables (Text S2); therefore, less causality information exists for the use of Tanh-based statistic than for the Skew-based statistic.

According to Table 3, Sleep problems were the estimated causal antecedent for depression as measured with the general-population oriented mBDI scale. Depression measured with the more clinically oriented BDI-II scale was the estimated cause of Sleep problems, but both DirectLiNGAM with the original non-standardized variables and the Tanh-based estimate were highly inconstent across the bootstrap re-samples. The general measure (i.e., DirectLiNGAM) indicated that sleep problems were a cause of mCES-D, whereas the Skew- and Tanh-based estimates contradicted this (Table 4). In the technical discussion that follows, the DirectLiNGAM results are nonetheless taken as the estimated causal directions for the three alternative pairings of sleep problem and depression variables; that is, Sleep problems cause mBDI, and BDI-II causes Sleep problems in the Young Finns data, and Sleep problems cause mCES-D in the Wisconsin Longitudinal Study data.

**Table 4:** Pairwise Causality Comparisons for 2000 Bootstrap Re-samples in the Data from Wisconsin Longitudinal Study.

	Chosen as cause %		Summary of values	
Method/Statistic	mCES-D	Sleepproblems	Statistic	95%confidence int.
DirectLiNGAM^a^	0.00	100.00	−0.8798	(−0.8940,−0.7940)
DirectLiNGAM^b^	0.00	100.00	−0.5655	(−0.6031,−0.5185)
Skew-based	100.00	0.00	0.0443	(0.0205,0.0730)
Tanh-based	99.85	0.15	0.0042	(0.0013,0.0071)

a non-standardized original variables.

b standardized variables; Skew- and Tanh-based statistic always require latter. Second and third column report the percentages of ‘wins’ in the indicated pairwise comparison, whereas the two last columns summarize the statistic implying the result over the 2000 re-samples. mCES-D = modified Center for Epidemiologic Studies Depression scale.

### Evaluation of LiNGAM Assumptions and Model Fit

**Figure 2:**
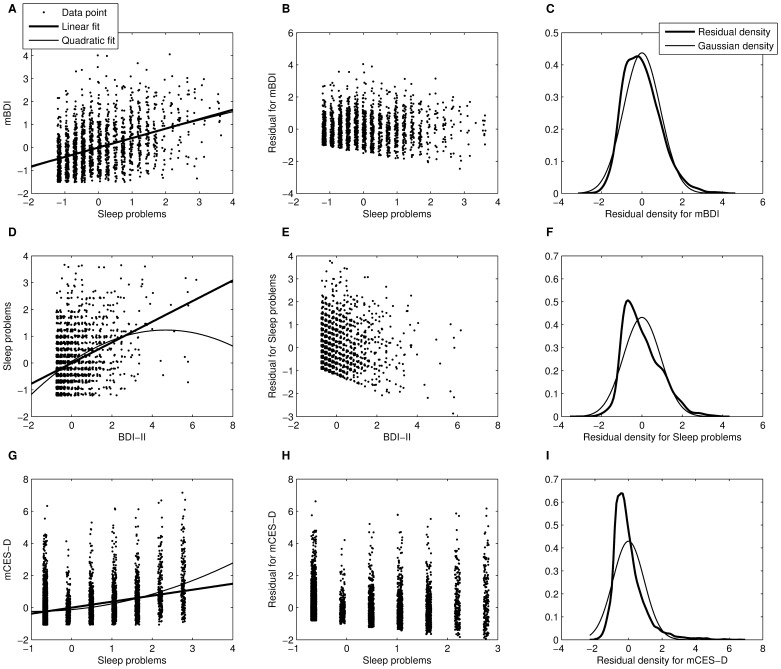
Three linear (Ordinary Least Squares) regression models corresponding to causal directions estimated by DirectLiNGAM-algorithm. Each row shows data for a model estimated in one data set. First panel of a row (A, D, or G) shows the linear (thick line) and quadratic (thin line) fits, superimposed on the data points. Jitter (a uniform random variable ranging from −0.1 to 0.1) was added to variables to enhance visibility of data points. Second panel is a scatterplot of the linear model residual against the independent variable. Last panel of each row shows a Gaussian probability density with mean and standard deviation equaling those of the observed residual distribution, and a kernel density estimate of the observed linear model residual.

After deriving the DirectLiNGAM causality estimates, we assessed whether the assumed models are fitting descriptions of the data for the recognized directions of causality. Figure 2 visually illustrates the linear model fit for each regression model, and the associated residual distributions. Table 5 collects P-values from statistical hypothesis tests for the existence of a quadratic (i.e., non-linear) term in regression model, for the non-Gaussian residual distribution, and for the dependence between the exogenous variable and residual.

**Table 5:** P-values for Statistical tests evaluating LiNGAM assumptions.

Estimated causal model	H_0_: β_quadratic_ = 0	H_0_: µ_e_ = Gaussian	H_0_: µ_X_×µ_e_	H^†^_0_: µ_X_×µ_e_
offspring’s SES = f(parents’ SES)+e	.013	<.001	1.05⋅10^−8^	.002
mBDI = f(Sleep problems)+e	.657	<.001	.079	.003
Sleep problems = f(BDI-II)+e	6.06⋅10^−6^	<.001	1.79⋅10^−16^	<10^−8^
mCES-D = f(Sleep problems)+e	7.37⋅10^−21^	<.001	<<.001	<10^−8^

Note: Leftmost column shows the evaluated model in the form where variable *Y* is a function *f* of *X* plus an error term *e*, denoted *Y = f(X) + e*. The second column is a p-value for the null-hypothesis that the *f* does not have quadratic nonlinearity, third column for the *e* being Normally distributed, fourth for the independence of *X* and *e* using L^1^-test, and final column tests independence by Hoeffding’s test (‘†’ superscripted). Lilliefor’s test for normality was based on tabulated values, and did not allow higher precision than given. For independence test between the residual and independent variable in the Wisconsin Longitudinal study data (n = 6640), very small p-value was obvious but exact value difficult to find using a standard function-minimizer.

First, visually the linear model seemed to be a fitting description when the estimated direction of causality was from Sleep problems to depressive tendencies assessed with mBDI. In the large WLS data (n = 6640; depression assessed with mCES-D) the quadratic term was statistically significant (Table 5), but the model was close to linear within the support of the data (Figure 2). In contrast, the nonlinear term was prominent between Sleep problems and BDI-II (Fig. 2 and Table 5). Second, the assumption of non-Gaussian distribution was satisfied for all data sets (Fig. 2 and Table 5). Third, and the most difficult, question is whether the ‘independent’ variable and residual term can be considered to be statistically independent of each other. Independence seems to be a reasonable approximation in the Young Finns data when modeling the outcome mBDI with the Sleep problems as an independent variable, although not strictly true (Table 5 and Figure 2); for other depression-sleep cases of Table 5, however, the Hoeffding’s *D*-statistic was 4 to 96 times larger than for the mBDI-outcome, indicating more dependency. The visual evaluation implied that the situation was not the worst possible in the large WLS data either, although the hypothesis of independence was strictly rejected. Also, a 1.4 times larger *D*-statistic for the benchmark SES model than for mBDI, and the significant quadratic coefficient (Table 5), did little to hinder the efficient causality estimation in the benchmark data (Table 3). For the model with BDI-II as independent variable and Sleep problems as dependent variable, the assumed independence clearly did not hold. Models are always approximations, and a simulation study further probed sensitivity to assumption violations.

### Simulation Study of Discretization and Confounding Effects

**Figure 3:**
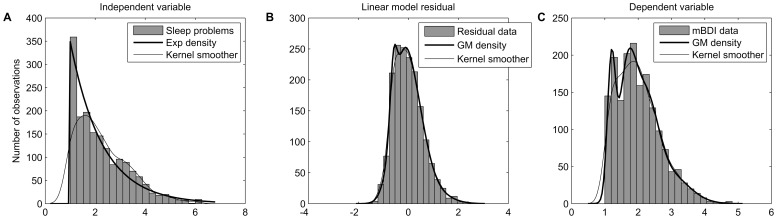
Simulation study approximating the observed data. The situation where mBDI was linearly modeled in the Young Finns data using Sleep problems as independent/predicting variable was modeled. Histograms of Sleep problems (A), Ordinary Least Squares residual of mBDI (B), and the dependent mBDI (C) are shown, together with probability distributions fitted to these data (thick lines, y-axis re-scaled for the number of observations), and (Gaussian-) kernel density estimates of the data (thin lines). First panel suggests that Mixture of Gaussians is not a good model for Sleep problems; a shifted Exponential distribution was chosen.

**Figure 4:**
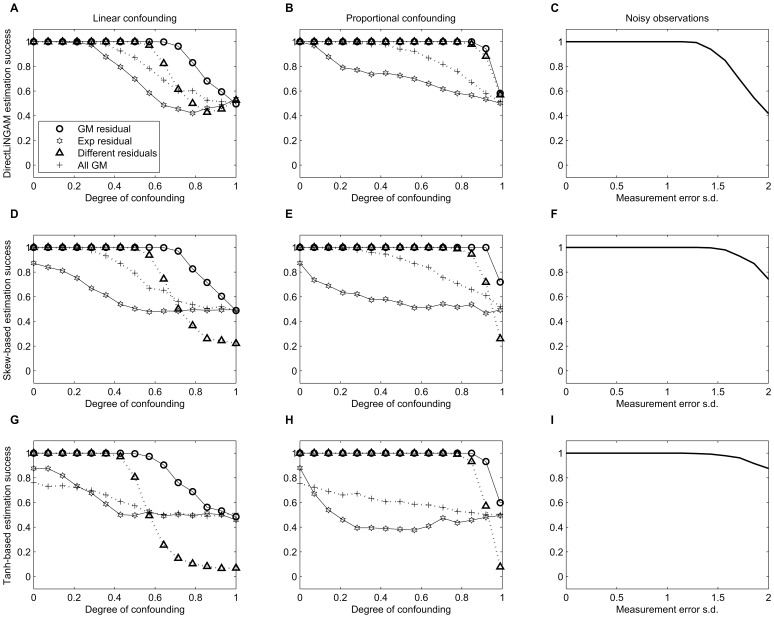
Simulation results by gradually perturbing the model of Figure 3. The rows signify the applied causality statistic: DirectLiNGAM-based (panels A,B,C), Skew-based (D,E,F), and Tanh-based statistic (G,H,I). Two leftmost panels of each row show estimation success (proportion of correct estimates) as a function of the degree of latent confounding. Different types of confounding (linear or proportional) and different distributional conditions were tested: Gaussian mixture (GM), Exponential (Exp), and GM and Exp (different) residual, and with all GM distributions; see methods. Last panel shows estimation success when an amount of Gaussian ‘measurement error’ indicated by horizontal axis was added to independent variable.

Figure 3 summarizes the constructed probability model that imitated the observed data during the simulations. The causality algorithms handled the discretization of data very well, indicating that ordinal variables should not be a problem provided that an underlying continuity exists: discretization down to just two categories yielded a 99.9% estimation success, and with 3 to 15 categories a flawless performance was observed for the LiNGAM- and Skew-based estimates; Tanh-based estimate only erred four out of thousand times for four-category variables, and twice for two-category variables. The algorithms also tolerated Gaussian measurement error in the exogenous variable very well: a performance decline was observed for the DirectLiNGAM-based estimate only after the measurement error variance exceeded the true variance of mBDI (i.e., 1.28^2^; see Figure 4C); the Skew-based estimate was even less sensitive (Fig. 4F), and the Tanh-based estimate appeared most robust against measurement noise (Fig. 4I).

Latent confounding was a more difficult question, and algorithm performance depended a lot on the underlying distributions and type of confounding (proportional/mixture vs. linear). Figure 4 indicates that a small amount of confounding was not a problem for the general estimate, but the Tanh- and Skew-based estimates were less robust against confounding; with some distribution settings and a large amount of confounding, they can be even biased. Partial confounding tended to disturb the causality estimation more when the residual distribution was a translated Exponential than when it was a mixture of four Gaussians. The relative performances of the three methods were mirror-images with respect to tolerance for confounding *versus* tolerance for measurement error: the Tanh-based estimate was the most noise-robust and least tolerant for confounding, and the DirectLiNGAM estimate obtained the opposite pattern. Since all measures tolerated quite a lot of measurement error, the potential confounding appears to be a more acute problem.

### Some Differences between the Depression Scales

**Figure 5:**
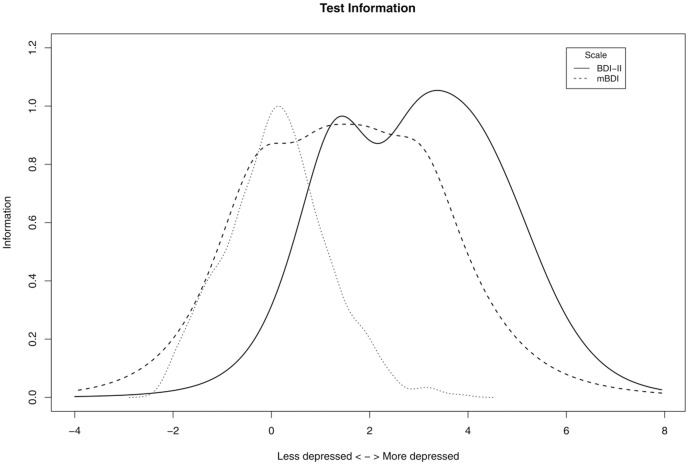
Total Test Information for the items of BDI-II (solid line) and for those of mBDI (dashed line). Units of the horizontal axis represent standard deviations of the latent/general depression as estimated by unidimensional Graded Response Model. Information per latent depression value holds no absolute meaning; it is estimated by integral over an adjacent step in 200 point discretization of horizontal axis. In addition to (Fisher) Information-content of the scales, the thin dotted line plots a Gaussian kernel density estimate from the factor scores of the estimated Graded Response Model, normalized to maximum of one; this serves to illustrate which severity-levels were actually present in the population-based Young Finns data set.

Despite the same Young Finns data, 22.8% of participants had answered ‘no symptom’ to all BDI-II items compared to only 1.5% in mBDI. For mCES-D, 5.7% of participants reported the lowest attainable score. This, and the different nonlinearities with respect to Sleep problems (Figure 2), suggested that despite their high correlation (r = 0.77) mBDI and BDI-II might differ with respect to some depression properties they measure. A Graded Response model was estimated in order to evaluate what relative information mBDI and BDI-II encode. Although the model did not fit well in the absolute sense (over half of the two-way item-margins indicated lack of fit for observed and expected frequencies of response patterns [51], [52]), in a relative sense, the model nonetheless indicated that the two measures did not encode fully overlapping information (Figure 5): mBDI encoded better than BDI-II for the lower levels of depression that were most present in this general-population sample. Scales that place a lot of weight on only severe depression may be problematic in population studies, as the study-attrition tends to associate with high depression scores (Tables 1 and 2). Indeed, the effect size (Cohen’s d) of the attrition on the depression score was three times larger for the BDI-II (d = 0.637) than for the population-oriented mBDI (d = 0.213) or mCES-D (d = 0.219) scales.

## Discussion

This study tested recently introduced causality estimators, that are able to estimate causality from cross-sectional data [1]–[3], on an epidemiological problem that can be considered truly open with respect to the issue of causality: does depression cause sleep problems in the general population, or vice versa? It was first shown that each of the three applied estimators easily recognized the correct causality from a benchmark data consisting of parents’ and their offspring’s socioeconomic status. The estimators quite consistently indicated that sleep problems caused depressive symptoms in one of the three data sets that best fulfilled the required assumptions for causality estimation (mBDI-data of the Young Finns study). In the same Young Finns data, another depression measure (BDI-II) yielded an inconsistent result, but this data set violated the assumptions of the model; the relationship was not linear, and the residual clearly depended on the independent/predictor variable value. In addition, the Wisconsin Longitudinal Study’s data violated one or both of these assumptions, although to a lesser degree, and provided conflicting results among the different estimators. A simulation study imitating present data characteristics revealed a dose-response relation between the degree of assumption violation and causal estimation-failure frequency. The DirectLiNGAM-based estimator in particular, that utilizes mutual information between regression residuals and independent variables, tolerated small violations in assumptions well. It also indicated the causal antecedence of Sleep problems in the Wisconsin data.

The results are partly in line with the causal implication of many studies that have found sleep problems to precede depression in time [23]–[27]; although compared to benchmark data and simulations, it appears that some amount of confounding and/or reciprocal effects exist between depression and sleep problems. Depression seemed to cause sleep problems according to a single DirectLiNGAM-comparison out of six, that for the standardized BDI-II variable. Non-standardized variable yielded less consistent results for BDI-II, which further undermines interpretation since the DirectLiNGAM should be invariant to standardization [2]; the lack of invariance may have resulted from the significantly nonlinear association between sleep problems and BDI-II (Figure 2). Hence, most results where the data appeared to follow the required LiNGAM assumptions to a reasonable degree indicated that the dominant direction of causality was from sleep problems to the depressive symptom score. Furthermore, depression scales for community-based studies (e.g., mBDI and mCES-D) tend to provide more information on lower degrees of depression severity than more clinically oriented scales, such as the unmodified version of Beck’s Depression Inventory [51]; a result that was supported by Graded Response Modeling of mBDI and BDI-II in the present study (Figure 5). The degree of depression severity may play a role in population association between depression and sleep problems.

There are two important ways for the degree of severity assessed by a scale to influence the results from LiNGAM estimates of causality. First, a measure like BDI-II appears to concentrate its informative range on severe depression [51], being relatively uninformative for a great number of mildly depressed participants in common-population samples (Figure 5); such a selective attenuation precludes the linear association that is required for causality estimation unless the sleep-problem covariate is also sensitive only for the same participants. A strong nonlinearity was indeed observed between Sleep problems and BDI-II in the Young Finns data, and 22.8% of participants had the lowest possible score in BDI-II compared to only 1.5% for the mBDI. Due to its emphasis on severe depression, the study attrition also had thrice the effect on BDI-II that it had on the mBDI and mCES-D. Second, the causal association in question may differ for severe and mild depression. For example, emerging evidence indicates that sleep problems induce immune system alterations, and immune dysfunction may be depressogenic [21]; also, severe depression itself causes major immune system alterations [21]. Another recent study showed that depressive symptoms rather form a causal network than reflect a single latent cause [30]; among the depressive symptoms, sleep problems are the prominent correlate of immune system alterations [21], [53]. From the causal network point of view, it is therefore plausible that sleep problems serve to initiate fatigue and mild depression through immune system dysfunction, whereas causality in severe depression is more mixed due to a large interconnected network of symptoms, life-events, and feedback from depressed mood to sleep and the immune system.

It has been shown that pairwise measures of causal association are robust against measurement noise or error [3], and the same was true for the computer simulation that imitated the present context. Our simulations also indicated that a small amount of confounding by an unobserved third variable did not necessarily preclude the estimation of causality with the DirectLiNGAM-based statistic, whereas the more approximate measures based on only certain kinds of departures from Normality did worse with respect to confounding. They did have the benefit of greater tolerance for symmetric measurement error, but DirectLiNGAM also had a surprisingly good tolerance for such errors. This dissociation between measurement- and confounding-related sensitivity may explain the dissociation in the causality estimates between Sleep problems and Depression that was observed for DirectLiNGAM-based and Tanh-/Skew-based estimates of the Wisconsin data (Table 4). The simulations suggest that differing results may be due to partial confounding/cyclicity for which Tanh- and Skew-based estimates are more sensitive than the DirectLiNGAM estimate. This would mean that while depression causes some Sleep problems in the Wisconsin data, the effect of Sleep problems on depression was greater still (i.e., only partial confounding). Interpreting this together with the results on mBDI and BDI-II scales, the logical implication is that the more severe depression one measures, the more it appears to inflict or confound with Sleep problems, but *for less severe depression*, sleep problems appear to serve as a causal antecedent. That is, Sleep problems are estimated as potent initiators of dysphoria or other depressive symptoms. Such a finding aligns with inferences made from the similarities between the patterns of neurobiological changes in chronic sleep deprivation and in depression: “chronic sleep deprivation may be a precursor of depression” [55].

The possibility of cyclic (reciprocal) causal relation between depression and sleep problems is intuitively sound and has been implicated in previously reported research [54], [56]. The LiNGAM-based approach is unable to learn such a model from the data. The confounding simulation suggested that if reciprocal causality were asymmetric, in the sense that one variable causes more of the other, the DirectLiNGAM-algorithm should detect the dominant cause. Although an algorithm for the estimation of a *cyclic*, linear, non-Gaussian, causal model does exist [4], it needs to rely on Independent Component Analysis [4], [32] rather than direct estimation methods [2], [3]. Causal methods based on Independent Component Analysis provide less reliable estimates than direct estimation methods [2], [3]. In addition, the authors of the method concluded that a “number of questions remain open” [4]. In addition, the cyclic method needs to assume that the underlying system is observed in an equilibrium state [4], whereas depression has a complex age-dependent biology [57], [58]. Therefore, our results only suggest a *dominant* causal flow from antecedent sleep problems to mild depressive symptomatology. Such findings are nonetheless important due to ongoing debate on lowering of thresholds for depression-like diagnoses and for initiation of their somatic therapy [59]; they are also of interest for the scientific understanding of depression etiology, as minor depression can be a transitional state on a path towards major depression [60].

Although research toward cyclic estimation may be beneficial for the understanding of depression-sleep connection, developing robust [61] versions of causality algorithms might be of a more immediate benefit. Depression is a multi-cause condition [62] with clearly established dependence on individual life-events [30], [63] as well as with the individual biology [21], [64]. Automated modeling of effects as being present in only part of the sample/population, as in robust statistics [61], has yielded benefits in complex psychobiological epidemiology [65]; many of the causal effects affecting majority of population might be more readily seen provided that less frequent routes to depression do not dilute them. Furthermore, it might be possible to alleviate the study attrition-based problems via some future missing-data models.

Regarding study limitations, it is not surprising that participants who were excluded due to lacking data had higher depression scores than the study samples (Tables 1 and 2), because inefficiency and lack of initiative are typical for depressed people. Equally unsurprisingly, women were over-represented in our data, as the men were more likely to lack data. Sleep problems and depression were self-reported by the same informant, resulting in possible common-rater variance. Future studies might measure these variables also with clinical interview of mental health and laboratory recordings of sleep, in addition to self-reports. A strength of the current study is the use of three depression measures, two sleep problem measures, two large populations, and three causality statistics. The reliability of the results was also evaluated by a numerical simulation using a setting that imitated the properties of the observed data, and by a benchmark data test.

In summary, this study provides one of the first applications of cross-sectional statistical estimation of pairwise causality to a challenging real-world epidemiological problem, as opposed to simulations and benchmark testing with ‘toy problems’. A prediction is drawn from these estimates for future empirical studies to confirm: for minor forms of depression and sensitive measures, sleep problems cause significantly more dysphoria/depression than dysphoria causes sleep problems; the situation changes as depression gets more severe, or more severe levels of symptoms are evaluated. It remains unclear as to whether the dominant causality becomes reversed or is balanced for more severe depression, and study attrition appears to present an increasingly severe problem for causality estimation in increasingly severe depression. This study is another piece of evidence for the causal role of sleep problems in the population-level etiology of depression, in addition to their temporal precedence [21]–[27] and physiological effects [18], [20]–[24], [54], [56].

## Supporting Information

Table S1 **Causality Estimates without the Removal of Sleep-related Items in Beck’s Depression Inventories.**
(DOC)

Text S1 **Supplementary Information on the Benchmark Analysis of Parents’ and Offspring’s Socioeconomic Status.**
(PDF)

Text S2 **Technical Information about the Three Causality Statistics.**
(PDF)

Demos S1 **Examples of Computations and Data Sets.**
(ZIP)
