# Supplementary material for: Pairwise Measures of Causal Direction in the Epidemiology of Sleep Problems and Depression
Source: PLoS One. 2012 Nov 30;7(11):e50841. doi: 10.1371/journal.pone.0050841 (PMC3511346; doi:10.1371/journal.pone.0050841)
Supplement: Table S1 — Causality Estimates without the Removal of Sleep-related Items in Beck’s Depression Inventories. (DOC) [file pone.0050841.s001.doc]

**Supplementary Table S1. Pairwise causality comparisons from 2000 bootstrap re-samples using all the 21 depression-items (i.e., sensitivity analysis for the effects of the removal of sleep-related items, cf. Table 3)**

|  | **Chosen as cause %** | | **Summary of values** | |
| --- | --- | --- | --- | --- |
| **Method/Statistic** | **mBDI** | **Sleep problems** | **Statistic** | **95% confidence int.** |
| DirectLiNGAM^a^ | 0.15 | 99.85 | -0.0453 | (-0.0756, -0.0093) |
| DirectLiNGAM^b^ | 1.70 | 98.30 | -0.0382 | (-0.0676, 0.0021) |
| Skew-based | 1.15 | 98.85 | -0.0343 | (-0.0646, -0.0075) |
| Tanh-based | 29.05 | 70.95 | -0.0012 | (-0.0054, 0.0028) |
|  | **BDI-II** | **Sleep problems** | **Statistic** | **95% confidence int.** |
| DirectLiNGAM^a^ | 70.60 | 29.40 | 0.0152 | (-0.0417, 0.0708) |
| DirectLiNGAM^b^ | 100.00 | 0.00 | 0.1385 | (0.0619, 0.2263) |
| Skew-based | 100.00 | 0.00 | 0.0892 | (0.0387, 0.1480) |
| Tanh-based | 50.85 | 49.15 | 0.0000 | (-0.0053, 0.0048) |

a) Non-standardized original variables. b) Standardized variables; Skew- and Tanh-based statistic always require standardization. Second and third column report the percentages of ‘wins’ in the indicated pairwise comparison, whereas the two last columns summarize the statistic implying the result over the 2000 re-samples. mBDI = modified Beck’s Depression Inventory; BDI-II = Beck’s Depression Inventory II.
