# Supplementary material for: Pairwise Measures of Causal Direction in the Epidemiology of Sleep Problems and Depression
Source: PLoS One. 2012 Nov 30;7(11):e50841. doi: 10.1371/journal.pone.0050841 (PMC3511346; doi:10.1371/journal.pone.0050841)
Supplement: Text S1 — Supplementary Information on the Benchmark Analysis of Parents’ and Offspring’s Socioeconomic Status. (PDF) [file pone.0050841.s002.pdf]

Supplementary material on benchmark analysis: Causal direction between parents' and offspring's socioeconomic status

Parents' socioeconomic status was defined in the Young Finns data as follows. Total income of the family was enquired from the parents in the year 1983, when the participants (offspring) themselves were 6-21 years old; income prior to taxes (but after the removal of investments necessary for the obtaining of the income) was recorder with an eight point precision: 1 = 'less than 10 000 mk' (Finnish marks, the euro currency did not enter circulation until the year 2002); 2 = '20 001 - 30 000 mk'; 3 = '30 001 - 40 000 mk'; 4 = '40 001 - 50 000 mk'; 5 = '50 001 - 60 000 mk'; 6 = '60 001 - 80 000 mk'; 7 = '80 001 - 100 000 mk'; and 8 = 'more than 100 000 mk'. Number of years of education until the year 1986 was recorded for mother and for father; participants lacking one or both parents were excluded from analysis, as were those lacking any of the required data. The level of education was also enquired from both parents with a seven point precision: 1 = 'elementary school unfinished'; 2 = 'elementary school'; 3 = 'middle school/part of high school'; 4 = 'high school'; 5 = college-level'; 6 = 'vocational/technical/business school'; 7 = 'university'. All the five variables were (z-score) standardized, summed, and standardized again, in order to form the parents' socioeconomic status (SES) variable.

Offspring's SES was defined as follows. Gross income of the participant was enquired with an eight point precision: 1 = '10 000€ or less'; 2 = '10.001 – 20.000€'; 3 = '20.001 – 30.000€'; 4 = '30.001 – 40.000€'; 5 = '40.001 – 50.000€'; 6 = '50.001 – 60.000€'; 7 = '60.001 – 70.000€'; and 8 = 'more than 70.000 €'. Years of education that a participant had undertaken until the year 2007 was recorded; the age of participants ranged from 30 to 45 years at that point. Also the level of education was recorded with an eight point precision: 1 = 'vocational school or any kind of course or

institute degree'; 2 = 'technical college', 3 = 'vocational high school'; 4 = 'university studies without degree'; 5 = 'bachelor's degree'; 6 = 'master's degree'; 7 = 'licentiate'; 8 = 'professor'. All the three variables were (z-score) standardized, summed, and standardized again, in order to form the offspring's SES variable.

Altogether 1348 (587 men and 761 women) participants had both parents and answers to all the required enquiries. The main manuscript provides the benchmark results from the estimation of causal direction between the parents' and offspring's SES for these participants/offspring. The below figure summarizes the histograms for these variables, and their scatterplot relationship.

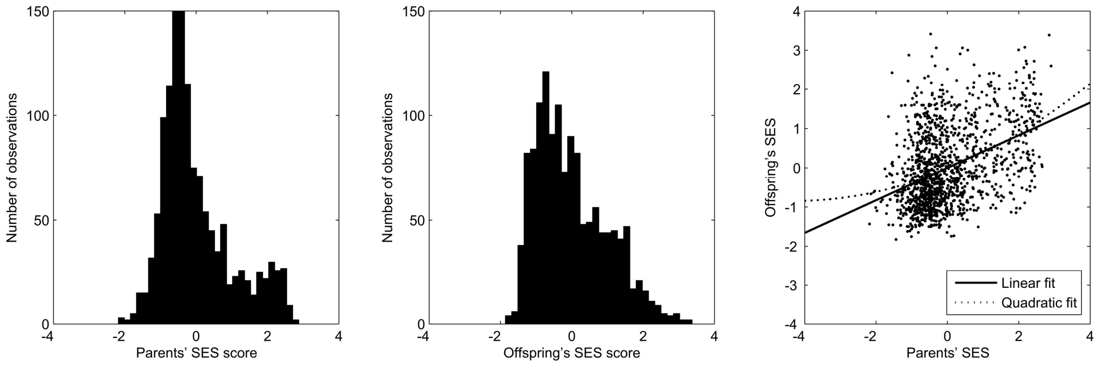

Supplementary Figure. Histograms and scatterplot of the parents' socioeconomic status (SES) and their offspring's SES. The linear and quadratic Ordinary Least Squares regression estimates are overlaid upon the scatterplot of the last panel.
