# Supplementary material for: Pairwise Measures of Causal Direction in the Epidemiology of Sleep Problems and Depression
Source: PLoS One. 2012 Nov 30;7(11):e50841. doi: 10.1371/journal.pone.0050841 (PMC3511346; doi:10.1371/journal.pone.0050841)
Supplement: Text S2 — Technical Information about the Three Causality Statistics. (PDF) [file pone.0050841.s003.pdf]

Supplementary material on the three causality statistics

This appendix details the three applied pairwise causality statistics. Recall that DirectLiNGAM performs two Ordinary Least Squares regressions: one with x_d as independent/predicting variable and x_s as outcome, and another with x_s as independent and x_d as the outcome. Then the causal antecedence is determined based-on which one is statistically less dependent of its residuals, x_d or x_s (see Methods). If we denote by $M(x_d,x_s)$ the mutual information between x_d and regression-residual of x_s and by $M(x_s,x_d)$ the mutual information between the opposite configuration, then under the LiNGAM assumptions the inequality $M(x_d,x_s) < M(x_s,x_d)$ implies that x_d is the causal antecedent and vice versa. Therefore, we can use the quantity

$$T(x_d,x_s) = M(x_s,x_d) - M(x_d,x_s)$$

as a causality statistic whose positive values indicate that x_d causes x_s, whereas the negative values indicate the opposite causality. Since we use the exact same kernel-based pairwise quantity $M(\cdot,\cdot)$ that the DirectLiNGAM-algorithm uses when deriving causal ordering of variables [2], we call this statistic T as the DirectLiNGAM-statistic; it aims to use general dependency information in variables. More restricted deviations from Gaussianity can also be used for the causality estimation.

The other statistics that function like T with respect to positive and negative values are the skewness- and kurtosis-based statistics. These use only some deviations from Gaussian distribution; namely, skewness and kurtosis. Let variables x_d and x_s be standardized (mean zero, variance one) and multiplied with the sign of their skewness (resulting in positive skewness), then the desired skewness-based statistics is

$$T_{skew}(x_d,x_s) = \rho(x_d,x_s)\mathrm{E}[x_d^2\, x_s - x_s^2 x_d],$$

where E is sample average or expectation, and $\rho(\cdot,\cdot)$ is the correlation of input variables. The below theorem establishes that under LiNGAM assumptions, a positive value of $T_{skew}(x_d,x_s)$ indicates that x_d is cause and a negative value indicates the antecedence of the second argument. The kurtosis-, or sparseness-based, statistic can also be derived, but it suffers from a lack of robustness and from sign-indeterminacy [3]. A hyperbolic tangent function (tanh) offers a more useful approximation [3, 32]. The explicit rationale is beyond present scope, but the ensuing statistic is

$$T_{tanh}(x_d,x_s) = \rho(x_d,x_s)\mathrm{E}[x_d \tanh(x_s) - x_s \tanh(x_d)],$$

where the input variables must be standardized. We call this the Tanh-based causality statistic.

T_{skew} and T_{tanh} apply only to standardized variables, but DiractLiNGAM-based statistic T can be applied to standardized and non-standardized variables; when everything goes according to assumptions, T should be invariant with respect to standardization [2]. Therefore we sometimes also provide results for both standardized and original variables in order to directly evaluate the sensitivity for scaling. For standardized random variables X_1, X_2, and X_3 the third cumulant, cum(X_1, X_2, X_3) = E[$X_1X_2X_3$], is multilinear (i.e., linear in each argument). Skewness of a standardized variable X is skew(X) = cum(X, X, X) [3, 32]. The following theorem is re-stated from previous work [3], and it proves that T_{skew} has the desired properties; that is, its sign implies the correct causality under the LiNGAM assumptions.

Theorem. *Let x and y be two standardized variables with positive skewness. If $y = \rho x + e$, with independent variables x and e and a constant coefficient ρ, then*

$$T_{skew}(x,y) = \text{skew}(x)(\rho^2 - \rho^3), \tag{1}$$

And if the causal direction is opposite, $x = \rho y + e$, then

$$T_{skew}(x,y) = \text{skew}(y)(\rho^3 - \rho^2). \tag{2}$$

Before proving the theorem, notice that variances of one for x and y force $|\rho| < 1$, and therefore the theorem implies that $T_{skew}(x,y)$ is positive when the first argument is cause and negative when the latter argument is the cause, provided the skewnesses of arguments are positive. If a variable x^* has a negative skewness, then the theorem can nonetheless be applied to $x = \text{sign}(\text{skew}(x^*))x^*$, which has a positive skewness. In practice, ρ is the usual correlation coefficient. Notice that skew(x) = 0 when x is a Gaussian variable.

Proof. Given the other assumptions and $y = \rho x + e$, we have

$$T_{skew}(x,y) = \rho(x,y)\text{E}[x^2y - xy^2]$$

$$= \rho[\text{cum}(x,x,\rho x + e) - \text{cum}(x,\rho x + e,\rho x + e)].$$

Because of multilinearity of the third cumulant, we obtain that the latter quantity is

$$\rho[\rho\text{cum}(x,x,x) + \text{cum}(x,x,e) - \rho^2\text{cum}(x,x,x) - 2\,\rho\text{cum}(x,x,e) - \text{cum}(x,e,e)]$$

$$= \rho[\rho\text{skew}(x) - \rho^2\text{skew}(x)] = \text{skew}(x)(\rho^2 - \rho^3).$$

This proves the equation 1, and equation 2 results from the symmetry $T_{skew}(y,x) = -T_{skew}(x,y)$. The second identity applied the fact that $\text{cum}(x,x,e) = \text{E}[x^2e] = \text{E}[x^2]\text{E}[e] =$

$0 = E[x]E[e^2] = E[xe^2] = \mathrm{cum}(x,x,e)$ for any square-integrable, standardized, and independent variables x and e.

///
